# Impact of Preoperative *vs* Postoperative Radiotherapy on Overall Survival of Locally Advanced Breast Cancer Patients

**DOI:** 10.3389/fonc.2021.779185

**Published:** 2021-11-23

**Authors:** Yujiao Deng, Hongtao Li, Yi Zheng, Zhen Zhai, Meng Wang, Shuai Lin, Yizhen Li, Bajin Wei, Peng Xu, Ying Wu, Xinyue Deng, Si Yang, Jun Lyu, Jingjing Hu, Huaying Dong, Zhijun Dai

**Affiliations:** ^1^ Department of Breast Surgery, The First Affiliated Hospital, College of Medicine, Zhejiang University, Hangzhou, China; ^2^ Department of Oncology, The 2^nd^ Affiliated Hospital of Xi’an Jiaotong University, Xi’an, China; ^3^ Department of Breast Head and Neck Surgery, The 3rd Affiliated Teaching Hospital of Xinjiang Medical University (Affiliated Tumor Hospital), Urumqi, China; ^4^ Department of Clinical Research, The First Affiliated Hospital of Jinan University, Guangzhou, China; ^5^ Dana-Farber Cancer Institute, Harvard Medical School, Boston, MA, United States; ^6^ Department of General Surgery, Hainan General Hospital, Hainan Affiliated Hospital of Hainan Medical University, Haikou, China

**Keywords:** locally advanced breast cancer, National Cancer Database, preoperative radiotherapy, postoperative radiotherapy, overall survival

## Abstract

**Background:**

The treatment for locally advanced breast cancer (LABC) is a severe clinical problem. The postoperative radiotherapy is a conventional treatment method for patients with LABC, whereas the effect of preoperative radiotherapy on outcome of LABC remains controversial. This study aimed to examine and compare the overall survival (OS) in patients with LABC who underwent preoperative radiotherapy or postoperative radiotherapy.

**Methods:**

This retrospective cohort study included 41,618 patients with LABC from the National Cancer Database (NCDB) between 2010 and 2014. We collected patients’ demographic, clinicopathologic, treatment and survival information. Propensity score was used to match patients underwent pre-operative radiotherapy with those who underwent post-operative radiotherapy. Cox proportional hazard regression model was performed to access the association between variables and OS. Log-rank test was conducted to evaluate the difference in OS between groups.

**Results:**

The estimated median follow-up of all included participants was 69.6 months (IQR: 42.84-60.22); 70.1 months (IQR: 46.85-79.97) for postoperative radiotherapy, 68.5 (IQR: 41.13-78.23) for preoperative radiotherapy, and 67.5 (IQR: 25.92-70.99) for no radiotherapy. The 5-year survival rate was 80.01% (79.56-80.47) for LABC patients who received postoperative radiotherapy, 64.08% (57.55-71.34) for preoperative radiotherapy, and 59.67% (58.60-60.77) for no radiotherapy. Compared with no radiation, patients receiving postoperative radiotherapy had a 38% lower risk of mortality (HR=0.62, 95%CI: 0.60-0.65, p<0.001), whereas those who received preoperative radiotherapy had no significant survival benefit (HR=0.88, 95%CI: 0.70-1.11, p=0.282). Propensity score matched analysis indicated that patients treated with preoperative radiotherapy had similar outcomes as those treated with postoperative radiotherapy (AHR=1.23, 95%CI: 0.88-1.72, p=0.218). Further analysis showed that in C0 (HR=1.45, 95%CI: 1.01-2.07, p=0.044) and G1-2 (AHR=1.74, 95%CI: 1.59-5.96, p=0.001) subgroup, patients receiving preoperative radiotherapy showed a worse OS than those who received postoperative radiotherapy.

**Conclusions:**

Patients with LABC underwent postoperative radiotherapy had improved overall survival, whereas no significant survival benefit was observed in patients receiving preoperative radiotherapy. Preoperative radiotherapy did not present a better survival than postoperative radiotherapy for LABC patients.

## Introduction

Breast cancer has become the most common cancer worldwide. Early breast cancer accounts for an increasing proportion of new breast cancer cases, and the disease burden continues to increase over time (1). Locally advanced breast cancer (LABC) encompasses stage III of the disease and a subset of patients with stage II (2), with a maximum lesion diameter of more than 5cm or lesion involving the surrounding skin or muscle, with or without axillary lymph node fusion and intramammary node, or ipsilateral supraconavicular node involvement.

The treatment of LABC is still a major challenge in patients with breast cancer because of the large space occupied by the primary lesions and serious local adhesions (3). Due to its low rate of overall survival (OS), high rate of recurrence and distant metastasis, LABC affects the overall survival of breast cancer largely (4). Currently, common adjuvant treatments for breast cancer are postoperative chemotherapy and radiotherapy (5). Radiotherapy is an effective treatment to reduce metastasis and improve the survival rate of breast cancer (6).

Recently, with the development of radiotherapy techniques, the value of preoperative radiotherapy has been reevaluated (7–10). Preoperative radiotherapy has been proven to prolong the prognosis of many cancers, such as rectal cancer (11), cervical cancer (12), et al. Some studies stated that preoperative radiotherapy could reduce the stage of tumor, increase the rate of surgical resection, alleviate symptoms and pain in patients, and improve the life quality of patients (9, 13). At present, there are few clinical studies on preoperative radiotherapy, and its effect for LABC patients is controversial (14–16). Early studies were mainly single-center, uncontrolled retrospective studies with small sample sizes, and the results were limited (17). In terms of long-term survival, the comparison between preoperative radiotherapy and postoperative radiotherapy lacks high-grade evidence-based data, and further investigation is needed.

The Nationally recognized National Cancer Database (NCDB), co-sponsored by the American College of Surgeons and the American Cancer Society, is a clinical oncology database derived from hospital registries collected by more than 1,500 Cancer Council accredited institutions. NCDB data were used to analyze and track patients with malignant cancer, their treatment and outcomes. The data represent more than 70 percent of newly diagnosed cancer cases and more than 34 million historical records nationwide (18). Based on the NCDB, we conducted this study to determine whether preoperative radiotherapy is superior to postoperative radiotherapy for the prognosis of patients with LABC. In this study, we analyzed the radiotherapy status of LABC patients who underwent surgery, and discussed the status and role of preoperative radiotherapy and postoperative radiotherapy in the treatment of LABC, as well as their prognostic value.

## Materials and Methods

### Study Design and Data Sources

We performed a retrospective review of the NCDB data of LABC patients diagnosed between January 1, 2010, and December 31, 2014. All adult women with LABC were selected by the ICD-O-3 (histological code <8800), and were assigned according to the 7^th^ AJCC TNM edition. Cases with LABC were defined as patients with stage III (T_0-2_N_2_M_0_, T_3-4_N_0-2_M_0_, T_0-4_N_3_M_0_) and part of stage II B (T_3_N_0_M_0_).

The inclusion criteria were as follows: (1) patients diagnosed with LABC in 2010-2014, microscopically confirmed, and only one malignant or *in situ* primary tumor in the patient’s lifetime; (2) patients who underwent breast surgery with a specific surgical procedure; (3) patients with no distant metastasis; (4) cases were females and aged ≥18.

We excluded cases for any of the following reasons: (1) lack of data on estrogen receptor, progesterone receptor, or human epidermal growth factor receptor 2 (Her-2); (2) unknown tumor grade or stage; (3) unknown status of chemotherapy, hormone therapy, or immunotherapy treatment; (3) lack of data on insurance, income, home location, vital status, or follow-up time; (4) if the patient received radiation therapy both before and after surgery or if they received intraoperative radiation with or without another therapy, in an unknow sequence except for postoperative radiotherapy, preoperative radiotherapy, and no radiation.

### Data Extraction and Outcomes

**Figure 1:**
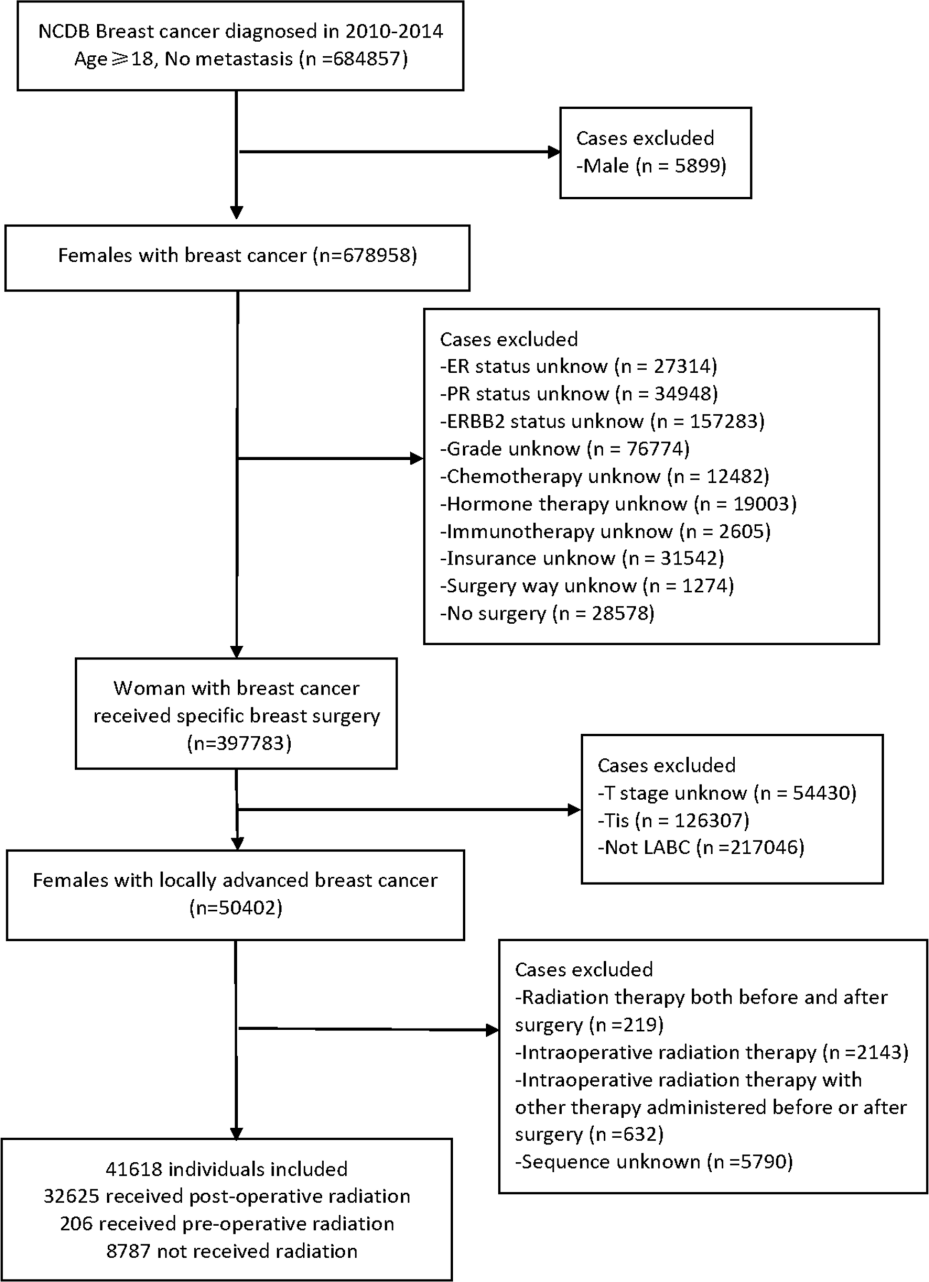
Flow Chart of participants Selection in National Cancer Database.

All included LABC patients were confirmed by cytology, histopathology, or microscopy and had only one lifetime history of malignancy or *in situ* recurrence, with no distant metastasis. We used the Charlson-Deyo Comorbidity Index (CCI) to quantify comorbid conditions. In total, eighteen factors were extracted: age at diagnosis, race, insurance provider (Medicaid, Medicare, or Private insurance/managed Care), median household income (high, high-middle, low-middle, or low), home location (rural, urban, or metro); CCI, grade (G1, well differentiated; G2, moderately differentiated; G3, poorly differentiated; G4, undifferentiated); tumor stage (T stage), nodal stage (N stage), molecular subtype (luminal, Her-2 positive, and triple-negative breast cancer); clinical stage, chemotherapy, hormone therapy, immunotherapy, surgery method; sequence of patients receiving radiotherapy and surgery, vital status, and follow-up time. The surgical procedure included total (simple) mastectomy, breast-conserving or -preserving surgery (BCS), and radical mastectomy. The race of the patients was divided into white, black, Asian/other. The pathological results of patients were classified into three categories based on ER, PR, and ERBB2 status. Luminal subtype was ER or PR positive, with or without ERBB2 positive. Her-2 positive subtype meant that both ER and PR are negative and ERBB2 is positive. Triple-negative subtype was defined as negative for estrogen receptor (ER), progesterone receptor (PR) and ERBB2 or Her-2. ER and PR were considered negative if less than 1% of cells stain positive. If the immunohistochemistry score was 0 to 1+ or fluorescence *in situ* hybridization and color *in situ* hybridization do not amplify, ERBB2 status was considered negative. The primary outcome was the rate of overall survival after breast surgery and radiotherapy. The endpoint was defined as the vital status of patients at last contact (alive or deceased). And the number of months to last contact were recorded. The diagram outlining all the selection criteria is presented in **Figure 1**.

### Statistical Analysis

We used frequency (percentage) to express categorical variables data and reported quantitative variables in quartile range (IQR). χ^2^ test or Fisher’s exact test was used for qualitative variables, and unpaired Kruskal-Wallis test was applied in quantitative variables. The Bonferroni test was conducted to compare sociodemographic, therapeutic, and tumor characteristics between the three treatment groups. In addition, from diagnosis to the last contact or death, the OS rate was calculated on a monthly basis. Univariate and multivariate Cox proportional hazard models were used to investigate the factors affecting OS in the unmatched and matched cohort. To solve the imbalance between patients receiving postoperative and preoperative radiotherapy, we conducted propensity score matching (PSM) analysis (19). We matched the conditional probability propensity scores for adjuvant radiotherapy before and after surgery. The variables included in the PSM analysis were age, race, insurance, income, home location, CCI, grade, T stage, N stage, molecular subtype, clinical stage, chemotherapy, hormone therapy, immunotherapy, surgery method. These variables are potential factors affecting the probability of receiving radiotherapy treatment. To avoid over-fitness, items (radiation and surgery sequence) entered into the PSM were excluded from the multivariate Cox regression analysis. The Kaplan-Meier curve was fit to calculate cumulative survival in unmatched and propensity matched cohorts. A log-rank test was performed to test the differences in the cumulative proportions across different treatment groups (20). Our study was reported followed the Strengthening the Reporting of Observational Studies in Epidemiology (STROBE) reporting guideline (**eTable 1**). All statistical tests were two-sided, the significance level of the Bonferroni test was 0.0167, and the significance level of other tests was 0.05. All statistical analyses were conducted using R software for Windows, version 4.0.5 (R Project for Statistical Computing).

## Results

### Patient Characteristics

A total of 41,618 cases met the inclusion criteria outlined above and were enrolled in our initial non-matched analysis (**Figure 1**). Among these patients, 32,625 (78.39%) experienced postoperative radiotherapy, 8,787 (21.11%) received no adjuvant radiation, and 206 (0.49%) endured preoperative radiotherapy. Compared with patients experienced preoperative radiotherapy, the postoperative radiotherapy cohort was younger (mean age, 59.24 *vs* 59.27, p<0.001), more Asians (p<0.001), more private insurance payers (p=0.002), more luminal tumors (p<0.001); and had better differentiation levels (p<0.001), lower tumor stage (p<0.001), higher nodal stage (p=0.005), better prognosis (p<0.001); more patients received hormone therapy (p<0.001) and BCS (p<0.001). There were no significant differences of distribution between preoperative and postoperative groups in income, home location, CCI grade, clinical stage, chemotherapy, and immunotherapy (**Table 1**).

**Table 1:** Patient demographic, disease, and treatment characteristics of locally advanced breast cancer grouped by radiation status.

Variable	Total population (No.)	No radiation		Postoperative radiotherapy		Preoperative radiotherapy		P value		
		(No.)	%	(No.)	%	(No.)	%	No radiation *vs* Postoperative radiotherapy	No radiation *vs* Preoperative radiotherapy	Postoperative radiotherapy *vs* Preoperative radiotherapy
**Age, mean (SD), years**	59.82(12.91)	60.98 (14.64)		59.24 (11.89)		56.27 (13.00)		<0.001^a^	<0.001^a^	<0.001^a^
**Age distribution (years)**										
<35	1415	187	13.22	1212	85.65	16	1.13	<0.001^a^	<0.001^a^	0.015^a^
35-50	10860	1575	14.50	9236	85.05	49	0.45			
50-70	20345	3434	16.88	16804	82.60	107	0.53			
≥70	8998	3591	39.91	5373	59.71	34	0.38			
**Race**										
White	33144	6927	20.90	26073	78.67	144	0.43	<0.001^a^	0.001a	<0.001^a^
Asia/other	2140	401	18.74	1731	80.89	8	0.37			
Black	6334	1459	23.03	4821	76.11	54	0.85			
**Insurance**										
Not insured	1436	267	18.59	1153	80.29	16	1.11	<0.001^a^	<0.001^a^	0.002^a^
Medicaid	4800	941	19.60	3830	79.79	29	0.60			
Medicare	12995	4258	32.77	8676	66.76	61	0.47			
Private Insurance/Managed Care	22387	3321	14.83	18966	84.72	100	0.45			
**Income**										
Low	7839	1937	24.71	5856	74.70	46	0.59	<0.001^a^	<0.001^a^	0.381
High	15333	2878	18.77	12384	80.77	71	0.46			
High-middle	9612	1980	20.60	7583	78.89	49	0.51			
Low-middle	8834	1992	22.55	6802	77.00	40	0.45			
**Home location**										
Rural/urban	5720	1207	21.10	4491	78.51	22	0.38	0.957	0.246	0.238
Metro	35898	7580	21.12	28134	78.37	184	0.51			
**Charlson Comorbidity Index**										
C0	34199	6684	19.54	27344	79.96	171	0.50	<0.001^a^	0.053	0.11
C1	5921	1574	26.58	4323	73.01	24	0.41			
C2-3	1498	529	35.31	958	63.95	11	0.73			
**Grade**										
G1-2	22435	4344	19.36	18008	80.27	83	0.37	<0.001^a^	0.0116^a^	<0.001^a^
G3-4	19183	4443	23.16	14617	76.20	123	0.64			
**Tumor stage**										
T0-1	7016	767	10.93	6224	88.71	25	0.36	<0.001^a^	<0.001^a^	<0.001^a^
T2	13610	2007	14.75	11551	84.87	52	0.38			
T3	17616	4828	27.41	12718	72.20	70	0.40			
T4	3376	1185	35.10	2132	63.15	59	1.75			
**Nodal stage**										
N0	8255	3153	38.20	5053	61.21	49	0.59	<0.001^a^	<0.001^a^	0.005^a^
N1	5787	1558	26.92	4198	72.54	31	0.54			
N2	19298	2771	14.36	16437	85.17	90	0.47			
N3	8278	1305	15.76	6937	83.80	36	0.43			
**Stage**										
S0-2	8050	2883	35.81	5134	63.78	33	0.41	<0.001^a^	<0.001^a^	0.988
S3-4	33568	5904	17.59	27491	81.90	173	0.52			
**Chemotherapy**										
No	8054	4207	52.23	3827	47.52	20	0.25	<0.001^a^	<0.001^a^	0.429
Yes	33564	4580	13.65	28798	85.80	186	0.55			
**Hormone therapy**										
No	12563	4408	35.09	8055	64.12	100	0.80	<0.001^a^	0.697	<0.001^a^
Yes	29055	4379	15.07	24570	84.56	106	0.36			
**Immunotherapy**										
No	39252	8457	21.55	30597	77.95	198	0.50	<0.001^a^	0.99	0.215
Yes	2366	330	13.95	2028	85.71	8	0.34			
**Subtype**										
Luminal	31257	5895	18.86	25247	80.77	115	0.37	<0.001^a^	<0.001^a^	<0.001^a^
Triple negative	7677	2209	28.77	5391	70.22	77	1.00			
Her-2	2684	683	25.45	1987	74.03	14	0.52			
**Surgery**										
Simple mastectomy	13582	3586	26.40	9938	73.17	58	0.43	<0.001^a^	<0.001^a^	<0.001^a^
BCS/other	9374	1200	12.80	8150	86.94	24	0.26			
Radical mastectomy	18662	4001	21.44	14537	77.90	124	0.66			
**Vital status**										
Alive	30352	5108	16.83	25116	82.75	128	0.42	<0.001^a^	0.28	<0.001^a^
Deceased	11266	3679	32.66	7509	66.65	78	0.69			

BCS, breast-conserving surgery; SD, standard deviation.

a Significance was evaluated using Bonferroni test. The statistical tests were two-sided, the significance level was 0.0167.

### Univariate and Multivariate Analysis

**Figure 2:**
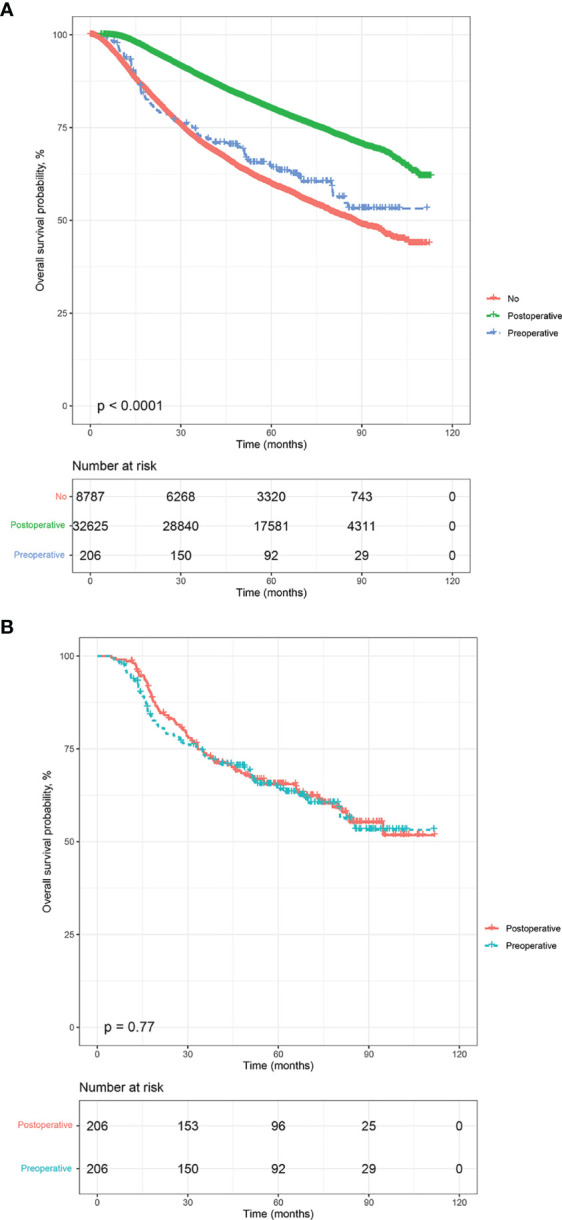
Kaplan-Meier overall survival analysis, before and after propensity score matching. **(A)** all participants, **(B)** matched population.

The estimated median follow-up time was 70.1 months (IQR: 46.85-79.97, range: 2.92-112.95, 95%CI: 69.7-70.5) for postoperative radiotherapy, 68.5 (IQR: 41.13-78.23, range: 4.99-111.57, 95%CI: 65.2- 74.8) for preoperative radiotherapy. The 5-year survival rate was 80.01% (79.56-80.47) for LABC patients receiving postoperative radiotherapy, 64.08% (57.55-71.34) for preoperative radiotherapy. In the survival analysis of the unmatched cohort, postoperative radiotherapy was related associated with improved OS compared to no radiation (p<0.0001) (**Figure 2A**). Similarly, in the multivariable Cox analysis adjusted for confounders, patients who received postoperative radiotherapy had a 38% lower risk of mortality [Adjusted HR (AHR) =0.62, 95%CI: 0.60-0.65, p<0.001]. However, there was no significant difference in prognosis between patients who received preoperative radiotherapy and those who did not (HR=0.85, 95%CI: 0.68-1.06, p=0.148; AHR=0.88, 95%CI: 0.70-1.11, p=0.282, **Table 2**).

**Table 2:** Univariable and multivariable Cox analysis of overall survival for patients with locally advanced breast cancer.

Variable	Total population	Alive	Deceased		Univariable Analysis		Multivariable Analysis	
		No.	No.	%	HR (95% CI)	P value	HR (95% CI)	P value
**Age distribution (years)**								
35-50	10860	8731	2129	19.60	1 (Ref.)		1 (Ref.)	
<35	1415	1055	360	25.44	1.36 (1.22-1.53)	<0.001	1.23 (1.10-1.37)	<0.001^a^
50-70	20345	15582	4763	23.41	1.22 (1.16-1.28)	<0.001	1.11 (1.05-1.17)	<0.001^a^
≥70	8998	4984	4014	44.61	2.80 (2.65-2.95)	<0.001	1.63 (1.52-1.76)	<0.001^a^
**Race**								
White	33144	24421	8723	26.32	1 (Ref.)			
Asia/other	2140	1758	382	17.85	0.68 (0.61-0.75)	<0.001	0.77 (0.69-0.85)	<0.001^a^
Black	6334	4173	2161	34.12	1.42 (1.36-1.49)	<0.001	1.16 (1.10-1.22)	<0.001^a^
**Insurance**								
Not insured	1436	1049	387	26.95	1 (Ref.)		1 (Ref.)	
Medicaid	4800	3416	1384	28.83	1.07 (0.95-1.19)	0.264	0.99 (0.89-1.11)	0.898
Medicare	12995	7975	5020	38.63	1.50 (1.35-1.66)	<0.001	0.97 (0.87-1.09)	0.646
Private Insurance/Managed Care	22387	17912	4475	19.99	0.66 (0.60-0.74)	<0.001	0.78 (0.70-0.86)	<0.001^a^
**Income**								
Low	7839	5336	2503	31.93	1 (Ref.)		1 (Ref.)	
High	15333	11767	3566	23.26	0.67 (0.63-0.70)	<0.001	0.94 (0.89-0.99)	0.041^a^
High-middle	9612	7020	2592	26.97	0.81 (0.76-0.85)	<0.001	0.97 (0.92-1.03)	0.344
Low-middle	8834	6229	2605	29.49	0.90 (0.85-0.95)	<0.001	1.03 (0.98-1.092)	0.27
**Home location**								
Rural/urban	5720	4041	1679	29.35	1 (Ref.)		1 (Ref.)	
Metro	35898	26311	9587	26.71	0.88 (0.84-0.93)	<0.001	0.93 (0.88-0.99)	0.014^a^
**Charlson Comorbidity Index**								
C0	34199	25771	8428	24.64	1 (Ref.)		1 (Ref.)	
C1	5921	3843	2078	35.10	1.54 (1.46-1.61)	<0.001	1.27 (1.21-1.33)	<0.001^a^
C2-3	1498	738	760	50.73	2.59 (2.41-2.79)	<0.001	1.67 (1.55-1.80)	<0.001^a^
**Grade**								
G1-2	22435	18100	4335	19.32	1 (Ref.)		1 (Ref.)	
G3-4	19183	12252	6931	36.13	2.18 (2.09-2.26)	<0.001	1.53 (1.46-1.60)	<0.001^a^
**Tumor stage**								
T0-1	7016	5454	1562	22.26	1 (Ref.)		1 (Ref.)	
T2	13610	9939	3671	26.97	1.25 (1.18-1.33)	<0.001	1.12 (1.05-1.19)	<0.001^a^
T3	17616	13198	4418	25.08	1.19 (1.12-1.26)	<0.001	1.57 (1.47-1.68)	<0.001^a^
T4	3376	1761	1615	47.84	2.75 (2.56-2.95)	<0.001	2.18 (2.02-2.36)	<0.001^a^
**Nodal stage**								
N0	8255	6478	1777	21.53	1 (Ref.)		1 (Ref.)	
N1	5787	4329	1458	25.19	1.18 (1.10-1.26)	<0.001	1.39 (1.24-1.54)	<0.001^a^
N2	19298	14438	4860	25.18	1.14 (1.08-1.21)	<0.001	2.35 (2.12-2.62)	<0.001^a^
N3	8278	5107	3171	38.31	1.90 (1.79-2.01)	<0.001	3.49 (3.14-3.89)	<0.001^a^
**Stage**								
S0-2	8050	6400	1650	20.50	1 (Ref.)		1 (Ref.)	
S3-4	33568	23952	9616	28.65	1.44 (1.37-1.52)	<0.001	1.14 (1.03-1.26)	0.012^a^
**Chemotherapy**								
No	8054	4794	3260	40.48	1 (Ref.)	<0.001	1 (Ref.)	
Yes	33564	25558	8006	23.85	0.49 (0.47-0.51)	<0.001	0.57 (0.54-0.60)	<0.001^a^
**Hormone therapy**								
No	12563	7024	5539	44.09	1 (Ref.)		1 (Ref.)	
Yes	29055	23328	5727	19.71	0.34 (0.33-0.35)	<0.001	0.62 (0.58-0.66)	<0.001^a^
**Immunotherapy**								
No	39252	28387	10865	27.68	1 (Ref.)			
Yes	2366	1965	401	16.95	0.69 (0.63-0.77)	<0.001	0.85 (0.77-0.95)	0.003^a^
**Subtype**								
Luminal	31257	24683	6574	21.03	1 (Ref.)		1 (Ref.)	
Triple negative	7677	3822	3855	50.21	3.34 (3.21-3.48)	<0.001	1.94 (1.81-2.09)	<0.001^a^
Her-2	2684	1847	837	31.18	1.60 (1.49-1.72)	<0.001	0.93 (0.84-1.02)	0.125
**Surgery**								
Simple mastectomy	13582	10482	3100	22.82	1 (Ref.)		1 (Ref.)	
BCS/other	9374	7279	2095	22.35	0.96 (0.91-1.01)	0.137	0.95 (0.90-1.01)	0.085
Radical mastectomy	18662	12591	6071	32.53	1.48 (1.42-1.55)	<0.001	1.12 (1.07-1.17)	<0.001^a^
**Radiotherapy**								
No	8787	5108	3679	41.87	1 (Ref.)		1 (Ref.)	
Postoperative radiotherapy	32625	25116	7509	23.02	0.43 (0.42-0.45)	<0.001	0.62 (0.60-0.65)	<0.001^a^
Preoperative radiotherapy	206	128	78	37.86	0.85 (0.68-1.06)	0.148	0.88 (0.70-1.11)	0.282

BCS, breast-conserving surgery; HR, hazard ratio.

a The statistical tests were two-sided, the significance level was 0.05.

Multivariable Cox analysis revealed that some factors were independently associated with improved or worse OS in LABC patients. Among these, the highest HR was for high nodal stage of LABC (N1/N2/N3 *vs*. N0), with N3 patients having an AHR of 3.49 (95%CI: 3.14-3.89, p<0.001, **Table 2**) and tumor stage ≥T2, and those with T4 having an AHR of 2.18 (95%CI: 2.02-2.36, p<0.001). Compared with well or moderately differentiated LABC, patients with poorly differentiated or undifferentiated histology had a 53% higher mortality risk (AHR= 1.53, 95%CI: 1.46-1.60, p<0.001). Compared with patients aged 35-50 years, patients aged <35 years, 50-70 years, and ≥70 years had a 23% (p<0.001), 11% (p<0.001), and 63% (p<0.001) higher mortality risk, respectively. Black patients had a 16% higher mortality risk (AHR= 1.16, 95%CI: 1.10-1.22, p<0.001) than white patients. Patients classified as C1 and C2-3 on the CCI had higher mortality risk values compared to C0 patients (C1: AHR=1.27, 95%CI: 1.21-1.33, p<0.001; C2-3: AHR=1.67, 95%CI: 1.55-1.80, p<0.001). Other factors associated with poor survival included clinical stage (stage 0-2 *vs* 3-4: AHR=1.14, 95%CI: 1.03-1.26, p=0.012), triple-negative subtype (triple-negative *vs*. luminal: AHR=1.94, 95%CI: 1.81-2.09, p<0.001), and the receipt of radical mastectomy (radical *vs*. simple: AHR=1.12, 95%CI: 1.07-1.17, p<0.001). In addition, some factors were associated with improved survival of patients with LABC. Asian and other races had a 23% lower mortality risk than white patients (AHR= 0.77, 95%CI: 0.69-0.85, p<0.001). Compared with patients who were not insured, private insurance payers had a 22% lower mortality risk (AHR= 0.78, 95%CI: 0.70-0.86, p<0.001). In addition, compared with low-income patients, those who carried a high median household income had a 6% lower mortality risk (AHR= 0.94, 95%CI: 0.89-0.99, p=0.041). Patients who lived in metro had a 7% lower mortality risk (AHR= 0.93, 95%CI: 0.88-0.99, p=0.014) than those who lived in rural or urban areas. As presented in **eTable 2**, patients who received preoperative radiotherapy combined with chemotherapy (HR= 0.34, 95%CI: 0.19-0.62, p<0.001) or hormone therapy (HR= 0.56, 95%CI: 0.36-0.88, p=0.012) showed better outcomes compared with their counterparts without corresponding treatments. The univariate Cox analysis results of patients who received postoperative radiotherapy were shown in **eTable 3**.

### Propensity Score–Matched Analysis and Outcomes

**Figure 3:**
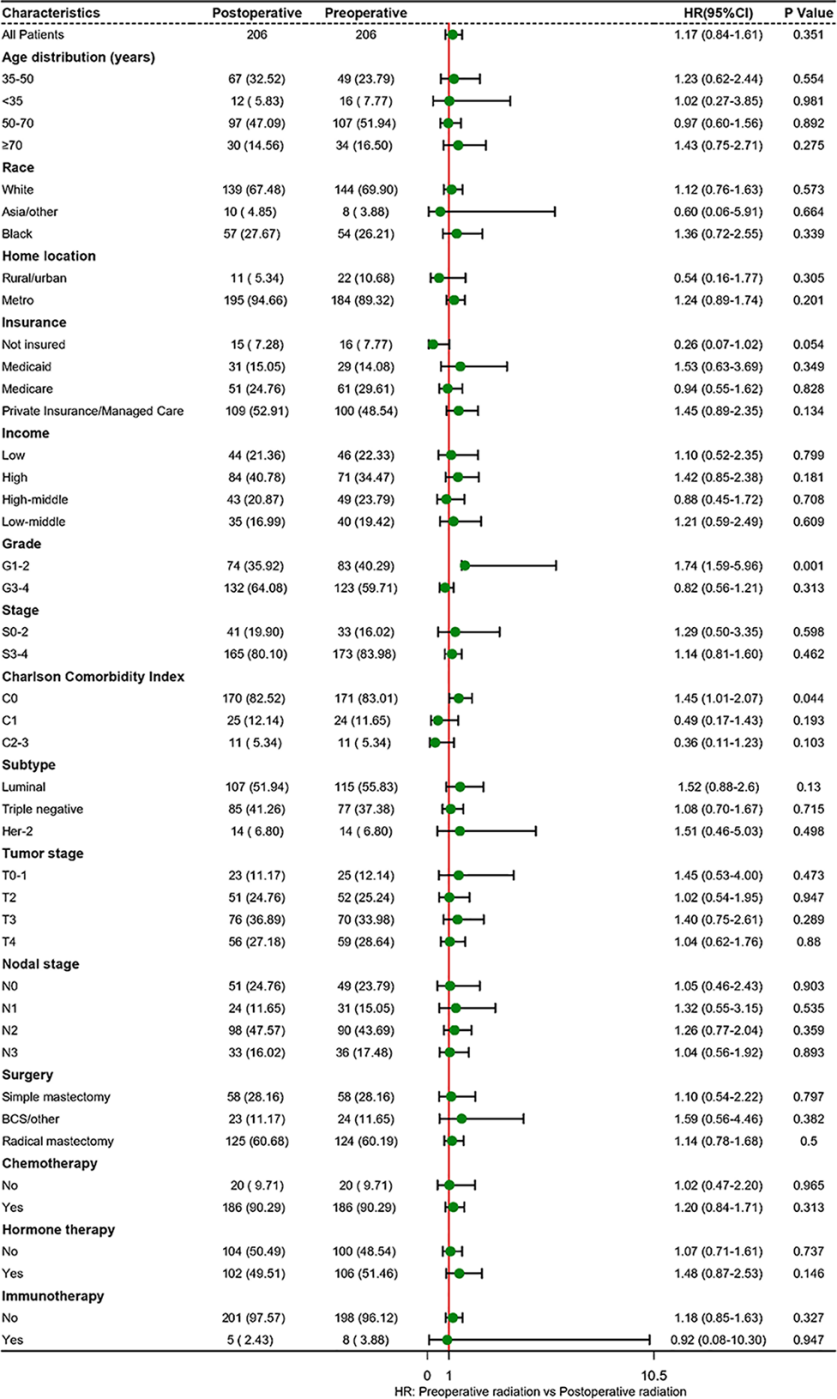
Subgroup analyses of radiotherapy treatment based on matched population. BCS, breast-conserving surgery; HR, hazard ratio.

The estimated median follow-up time was 71.4 months (IQR: 34.37-75.22, range: 4.50-107.04, 95%CI: 67.40-75.20) for patients who received postoperative radiotherapy and 68.5 months (IQR: 65.20-74.80, range: 4.99-111.57, 95%CI: 65.2- 74.8) for those who experienced preoperative radiotherapy. The 5-year survival rate was 66.29% (59.82-73.47) for those who received postoperative radiotherapy and 64.08% (57.55-71.34) for those who endured preoperative radiotherapy. In the multivariable analysis of the matched cohort (**Table 3**), patients aged ≥70 years had a three times higher risk of mortality (AHR= 3.83, 95%CI: 1.81-8.11, p<0.001) compared to those aged 35-50 years. Black patients had a 59% worse OS (AHR= 1.59, 95%CI: 1.07-2.37, p<0.001) than white patients. In addition, factors associated with poor OS in the matched cohort included tumor stages T3 (T3 *vs* T0-1: AHR= 2.09, 95%CI: 1.09-4.02, p=0.027) and T4 (T4 *vs* T0-1:AHR= 3.45, 95%CI: 1.82-6.54, p<0.001), nodal stages N1 (N1 *vs* N0: AHR= 3.37, 95%CI: 1.48-7.68, p=0.004), N2 (N2 *vs* N0: AHR= 10.01, 95%CI: 4.59-21.83, p<0.001), and N3 (N3 *vs* N0: AHR= 10.26, 95%CI: 4.62-22.78, p<0.001), triple-negative subtype (Triple negative *vs* Luminal: AHR= 9.02, 95%CI: 3.90-20.86, p<0.001), Her-2 positive subtype (Her-2 positive *vs* Luminal: AHR= 4.17, 95%CI: 1.48-11.72, p=0.007), and patients underwent radical mastectomy (AHR= 1.71, 95%CI: 1.10-2.66, p=0.017). Finally, patients who endured preoperative radiotherapy had a statistically similar prognosis to those who received postoperative radiotherapy (AHR=1.23, 95%CI: 0.88-1.72, p=0.218). Survival analysis indicated no difference existed in the OS of LABC patients between preoperative radiotherapy and postoperative radiotherapy (p=0.77, **Figure 2B**). In addition, patients in C0 (HR=1.45, 95%CI: 1.01-2.07, p=0.044) and G1-2 subgroup (AHR=1.74, 95%CI: 1.59-5.96, p=0.001) experienced preoperative radiotherapy showed a worse OS than those who received postoperative radiotherapy (**Figure 3**).

**Table 3:** Propensity-adjusted multivariable Cox regression analysis of overall survival for locally advanced breast cancer.

Variable	HR (95% CI)	P value
**Age distribution (years)**		
35-50	1 (Ref.)	
<35	1.09 (0.54-2.21)	0.814
50-70	1.33 (0.83-2.14)	0.233
≥70	3.83 (1.81- 8.11)	<0.001^a^
**Race**		
White	1 (Ref.)	
Asia/other	1.31 (0.45-3.81)	0.625
Black	1.59 (1.072-2.37)	0.021^a^
**Insurance**		
Not insured	1 (Ref.)	
Medicaid	1.27 (0.48-3.35)	0.63
Medicare	0.67 (0.26-1.74)	0.406
Private Insurance/Managed Care	1.09 (0.44-2.67)	0.858
**Income**	1 (Ref.)	
Low	1.35 (0.80-2.27)	0.259
High	1.09 (0.64-1.88)	0.741
High-middle	1.70 (0.99-2.90)	0.054
Low-middle		
**Home location**		
Rural/urban	1 (Ref.)	
Metro	1.13 (0.59-2.19)	0.71
**Charlson Comorbidity Index**		
C0	1 (Ref.)	
C1	1.12 (0.62-2.02)	0.706
C2-3	1.48 (0.74-2.97)	0.266
**Grade**		
G1-2	1 (Ref.)	
G3-4	1.07 (0.72-1.60)	0.736
**Tumor stage**		
T0-1	1 (Ref.)	
T2	1.76 (0.96-3.25)	0.07
T3	2.09 (1.09-4.02)	0.027^a^
T4	3.45 (1.82-6.54)	<0.001^a^
**Nodal stage**		
N0	1 (Ref.)	
N1	3.37 (1.48-7.68)	0.004^a^
N2	10.01 (4.59-21.83)	<0.001^a^
N3	10.26 (4.62-22.78)	<0.001^a^
**Stage**		
S0-2	1 (Ref.)	
S3-4	0.71 (0.30-1.68)	0.436
**Chemotherapy**		
No	1 (Ref.)	
Yes	1.06 (0.58-1.94)	0.855
**Hormone therapy**		
No	1 (Ref.)	
Yes	1.60 (0.71-3.58)	0.254
**Immunotherapy**		
No	1 (Ref.)	
Yes	1.46 (0.52-4.13)	0.472
**Subtype**		
Luminal	1 (Ref.)	
Triple negative	9.02 (3.90-20.86)	<0.001^a^
Her-2	4.17 (1.48-11.72)	0.007^a^
**Surgery**		
Simple mastectomy	1 (Ref.)	
BCS/other	1.80 (0.89-3.62)	0.1
Radical mastectomy	1.71 (1.10-2.66)	0.017^a^
**Radiotherapy**		
Postoperative radiotherapy	1 (Ref.)	
Preoperative radiotherapy	1.23 (0.88-1.72)	0.218

BCS, breast-conserving surgery; HR, hazard ratio.

a The statistical tests were two sided, the significance level was 0.05.

## Discussion

In this hospital-based registry analysis, postoperative radiotherapy presented a significant benefit for improved OS of LABC patients compared to no radiation, which appears to be consistent with a previous study (21). The benefit was also observed in patients who endured preoperative radiotherapy. However, the benefit was not statistically significant. PSM matched analysis indicated that, compared with postoperative radiotherapy, no survival improvement was observed in LABC patients who experienced preoperative radiotherapy. The effect of postoperative radiotherapy for LABC patients had been confirmed by several large clinical trials, which could significantly increase the local control rates and improve their OS rates (22).

In recent years, the value of preoperative radiotherapy in the treatment of LABC patients has been reassessed. Studies showed that new adjuvant chemotherapy improved the pathological complete response of tumors (23). A Previous study reported on the benefits of preoperative radiotherapy or chemotherapy on tumor treatment (24) and the impact of breast reconstruction surgery, as well as its value in tumor biology and translational medicine research. Our analysis illustrated that patients receiving preoperative radiotherapy combined with chemotherapy or hormone therapy showed prognosis benefit, which is consistent with published studies (25, 26). Through the combined use of preoperative radiotherapy and drugs, clinicians can obtain a clinical effect evaluation in a relatively short period of time and guide follow-up treatment by observing lesion changes (27, 28). However, approximately 1/3 of LABC patients are resistant to neoadjuvant chemotherapy, and there is still no manual resection opportunity for the tumor after chemotherapy. In this case, preoperative radiotherapy (21) or preoperative concurrent chemoradiotherapy is an important salvage treatment measure which could reduce the tumor load in some patients and provide the opportunity for surgical resection (2). Preoperative radiotherapy could increase the sensitivity of radiotherapy (29), cause tumor tissue fibrosis, reduce the risk of intraoperative implantation and metastasis, change the tumor microenvironment, transform the tumor immune escape state into a tumor immune attack state, and activate the immune system to produce long-distance effects (9, 30). However, the high incidence of acute toxic reactions is attributed to the lack of therapeutic experience and/or technical limitations due to factors such as concurrent chemotherapy, a high total dose of radiotherapy, and the limit of radiation techniques. Severe toxic reactions are the most important reason for the limited clinical application of preoperative radiotherapy or preoperative neoadjuvant concurrent chemoradiotherapy (31). Several studies have demonstrated the favorable effect of preoperative radiotherapy on tumor treatment and breast reconstruction surgery, as well as its value in tumor biology and translational medicine research.

Radiotherapy is important for the treatment of breast cancer, improving the local control rate and OS of patients at a high risk of recurrence. For advanced breast cancer (16), preoperative radiotherapy can reduce tumor stage, increase the resection rate, and alleviate the symptoms of patients.

Clinically, the selection of neoadjuvant radiotherapy for patients is limited to a certain extent, and there is currently no unified standard. Most clinical decisions depend on the clinical experience of doctors, so there may be the possibility of overtreatment. In our analysis, black patients with LABC were more inclined to endure preoperative radiotherapy, especially for patients with T4 stage tumors, aged 50-70 years, uninsured, triple negative subtype, poorly or undifferentiated. As for surgery method, the proportion of patients undergoing radical breast cancer resection undergoing preoperative radiotherapy was higher than that of patients undergoing other surgical procedures. Besides, neoadjuvant radiotherapy or chemoradiotherapy may lead to vascular injury and microcirculation disturbance, resulting in tissue cell degeneration and necrosis, breast fibrosis and skin injury. However, the fibrotic and damaged skin of the breast increases the difficulty of operation and prolongs the operation time, making radiotherapy as a neoadjuvant therapy method not widely employed for breast cancer (7). Suitable and safe treatment plans timelines, and treatment modalities with long survival rates, short and convenient reconstruction processes, and good appearance should be determined for LABC patients. In addition, biomarkers that are sensitive to radiation and chemotherapy should be ascertained.

A study based on 129,692 patients supported that breast-conserving surgery with radiation therapy improved the survival of breast cancer patients (26). Patients with stage IIB-IIIA breast cancer are generally considered having “operable breast cancer”. In contrast, those receiving postoperative radiotherapy or with stage IIIB and IIIC cancer are likely to be classified as inoperable cases; this is due to the presence of inflammation and/or extensive skin involvement, immobilization, or very large axillary lymph node disease, and/or the involvement of supraclavicular or internal breast lymph nodes (32). However, preoperative radiotherapy provides LABC patients with no chance of surgery with the opportunity of surgical treatment, as well as the opportunity of breast-conserving surgery for patients who cannot initially undergo breast-conserving surgery (24), thus improving their quality of life (33). By comparing the tumor tissues before and after radiotherapy and analyzing the various differences at the molecular level, biological information related to the radio sensitivity of tumor cells can be obtained, which helps to understand the changes in the immune microenvironment (34).

There are some limitations inevitable in this study. A small percentage of patients with LABC received preoperative radiation. Due to the limited data, we could not perform further subgroup analysis on the radiotherapy duration and dose of patients. The study population included patients who were diagnosed with LABC and underwent breast surgery. It should be emphasized that the application of these results cannot be expanded to general breast cancer. Although we used a retrospective paired study to select the control group, there is still an unavoidable selection bias, and there are some unknown influencing factors that will affect the final study conclusion. Besides, due to the limited data of preoperative radiotherapy, we had not been able to do a preoperative and postoperative analysis of other treatments (e.g., chemotherapy, endocrine therapy, immunotherapy) in LABC patients. However, we had enrolled those potential factors into the PSM analysis, and the effect of the variables has been largely balanced. In addition, we analyzed the relationship between the two types of radiotherapy combined with other treatments independently. We recommend that patients with LABC be treated in combination with chemotherapy or hormone therapy, regardless of preoperative or postoperative radiotherapy. Nevertheless, the role and value of preoperative radiotherapy or concurrent radio-chemotherapy for the treatment of LABC under the application of novel radiotherapy technologies and medicines requires confirmation and investigation by prospective, multi-center, randomized controlled clinical studies with large sample sizes.

In this study, patients with LABC who received postoperative radiotherapy were associated with improved OS, while those who received preoperative radiotherapy had no significant benefit. In the matched analysis, there was no significant difference in survival between patients receiving postoperative radiotherapy and those who receiving preoperative radiotherapy. The conclusions still need to be confirmed in large prospective clinical trials.

## Data Availability Statement

The original contributions presented in the study are included in the article/**Supplementary Material**. Further inquiries can be directed to the corresponding authors.

## Ethics Statement

The studies involving human participants were reviewed and approved by The First Affiliated Hospital of Zhejiang University. Written informed consent for participation was not required for this study in accordance with the national legislation and the institutional requirements.

## Author Contributions

All authors read, critically reviewed and approved the final manuscript. ZD and HD designed the research. YD, HL, JL, and ZZ collected the data. MW and SL verified the accuracy of the data. YD, SY, YL, and BW performed the statistical analysis. PX, YW, JH, and XD conducted the visualization. YD wrote the manuscript. YD and HL contributed equally to this work.

## Conflict of Interest

The authors declare that the research was conducted in the absence of any commercial or financial relationships that could be construed as a potential conflict of interest.

The reviewer QT has declared a shared parent affiliation with the authors MW, SL, PX, and YW at the time of review.

## Publisher’s Note

All claims expressed in this article are solely those of the authors and do not necessarily represent those of their affiliated organizations, or those of the publisher, the editors and the reviewers. Any product that may be evaluated in this article, or claim that may be made by its manufacturer, is not guaranteed or endorsed by the publisher.

## Supplementary Material

Supplementary Table 1 Strengthening the Reporting of Observational Studies in Epidemiology (STROBE) Reporting Guideline Checklist.

Supplementary Table 2 Univariate cox analysis of overall survival in patients with locally advanced breast cancer received preoperative radiation.

Supplementary Table 3 Univariate cox analysis of overall survival in patients with locally advanced breast cancer received postoperative radiation.

## Abbreviations

LABC, locally advanced breast cancer; OS, overall survival; NCDB, National Cancer Database; Her-2, human epidermal growth factor receptor 2; BCS, breast-conserving or -preserving surgery; IQR inter-quartile range; PSM, propensity score matching; AHR, adjusted hazard ratio.
