# Supplementary material for: Impact of Preoperative vs Postoperative Radiotherapy on Overall Survival of Locally Advanced Breast Cancer Patients
Source: Front Oncol. 2021 Nov 23;11:779185. doi: 10.3389/fonc.2021.779185 (PMC8650152; doi:10.3389/fonc.2021.779185)
Supplement: Supplementary Table 2 — Univariate cox analysis of overall survival in patients with locally advanced breast cancer received preoperative radiation. [file Table_2.docx]

**eTable 2. Univariate cox analysis of overall survival in patients with locally advanced breast cancer received preoperative radiation.**

| **Variable** | **Univariate analysis HR (95% CI)** | **P value** |
| --- | --- | --- |
| **Age distribution (years)** |  |  |
| 35-50 | 1(Ref.) |  |
| <35 | 1.04(0.38-2.86) | 0.941 |
| 50-70 | 1.11(0.60-2.03) | 0.745 |
| ≥70 | 3.50(1.82-6.71) | <0.001^a^ |
| **Race** |  |  |
| White | 1(Ref.) |  |
| Asia/other | 0.38(0.05-2.72) | 0.332 |
| Black | 1.06(0.64-1.75) | 0.82 |
| **Insurance** |  |  |
| Not insured | 1(Ref.) |  |
| Medicaid | 2.40(0.67-8.62) | 0.178 |
| Medicare | 3.20(0.97-10.54) | 0.056 |
| Private Insurance/Managed Care | 2.18(0.67-7.07) | 0.196 |
| **Income** |  |  |
| Low | 1(Ref.) |  |
| High | 1.60(0.84-3.02) | 0.151 |
| High-middle | 1.18(0.58-2.39) | 0.65 |
| Low-middle | 1.58(0.78-3.21) | 0.21 |
| **Home location** |  |  |
| Rural/urban | 1(Ref.) |  |
| Metro | 1.51(0.66-3.48) | 0.329 |
| **Charlson Comorbidity Index** |  |  |
| C0 | 1(Ref.) |  |
| C1 | 0.47( 0.19-1.18) | 0.108 |
| C2-3 | 0.95(0.34-2.59) | 0.913 |
| **Grade** |  |  |
| G1-2 | 1(Ref.) |  |
| G3-4 | 0.97(0.62-1.53) | 0.91 |
| **Tumor stage** |  |  |
| T0-1 | 1(Ref.) |  |
| T2 | 0.99(0.46- 2.13) | 0.979 |
| T3 | 0.83(0.39-1.76) | 0.633 |
| T4 | 1.53(0.74-3.18) | 0.246 |
| **Nodal stage** |  |  |
| N0 | 1(Ref.) |  |
| N1 | 2.17(0.95-4.93) | 0.065 |
| N2 | 1.95(0.99-3.85) | 0.055 |
| N3 | 3.78(1.81-7.87) | <0.001^a^ |
| **Stage** |  |  |
| S0-2 | 1(Ref.) |  |
| S3-4 | 1.84(0.92-3.68) | 0.087 |
| **Chemotherapy** |  |  |
| No | 1(Ref.) |  |
| Yes | 0.34(0.19-0.62) | <0.001^a^ |
| **Hormone therapy** |  |  |
| No | 1(Ref.) |  |
| Yes | 0.56(0.36-0.88) | 0.012^a^ |
| **Immunotherapy** |  |  |
| No | 1(Ref.) |  |
| Yes | 0.64(0.16-2.60) | 0.531 |
| **Subtype** |  |  |
| Luminal | 1(Ref.) |  |
| Triple negative | 2.30(1.45-3.66) | <0.001^a^ |
| Her-2 | 1.69(0.71-4.04) | 0.237 |
| **Surgery** |  |  |
| Simple mastectomy | 1(Ref.) |  |
| BCS/other | 1.46(0.64-3.30) | 0.366 |
| Radical mastectomy | 1.69(0.97-2.96) | 0.066 |

Abbreviations: BCS, breast-conserving surgery.

^a^ The statistical tests were two-sided, the significance level was 0.05.
