# Supplementary material for: Impact of Preoperative vs Postoperative Radiotherapy on Overall Survival of Locally Advanced Breast Cancer Patients
Source: Front Oncol. 2021 Nov 23;11:779185. doi: 10.3389/fonc.2021.779185 (PMC8650152; doi:10.3389/fonc.2021.779185)
Supplement: Supplementary Table 3 — Univariate cox analysis of overall survival in patients with locally advanced breast cancer received postoperative radiation. [file Table_3.docx]

**eTable 3. Univariate cox analysis of overall survival in patients with locally advanced breast cancer received postoperative radiation**

| **Variable** | **Univariate analysis HR (95% CI)** | **P value** |
| --- | --- | --- |
| **Age distribution (years)** |  |  |
| 35-50 | 1(Ref.) |  |
| <35 | 1.38(1.22-1.56) | <0.001^a^ |
| 50-70 | 1.20(1.13-1.27) | <0.001^a^ |
| ≥70 | 2.29(2.15-2.44) | <0.001^a^ |
| **Race** |  |  |
| White | 1(Ref.) |  |
| Asia/other | 0.77(0.68-0.86) | <0.001^a^ |
| Black | 1.52(1.44-1.61) | <0.001^a^ |
| **Insurance** |  |  |
| Not insured | 1(Ref.) |  |
| Medicaid | 1.04(0.91-1.19) | 0.543 |
| Medicare | 1.28(1.14-1.45) | <0.001^a^ |
| Private Insurance/Managed Care | 0.67(0.60-0.76) | <0.001^a^ |
| **Income** |  |  |
| Low | 1(Ref.) |  |
| High | 0.67(0.63-0.72) | <0.001^a^ |
| High-middle | 0.77(0.72-0.83) | <0.001^a^ |
| Low-middle | 0.85(0.79-0.91) | <0.001^a^ |
| **Home location** |  |  |
| Rural/urban | 1(Ref.) |  |
| Metro | 0.90(0.84-0.96) | 0.001^a^ |
| **Charlson Comorbidity Index** | |  |
| C0 | 1(Ref.) |  |
| C1 | 1.48(1.39-1.57) | <0.001^a^ |
| C2-3 | 2.39(2.16-2.64) | <0.001^a^ |
| **Grade** |  |  |
| G1-2 | 1(Ref.) |  |
| G3-4 | 2.20(2.10-2.30) | <0.001^a^ |
| **Tumor stage** |  |  |
| T0-1 | 1(Ref.) |  |
| T2 | 1.18(1.10-1.26) | <0.001^a^ |
| T3 | 1.08(1.01-1.16) | 0.025^a^ |
| T4 | 2.40(2.20-2.62) | <0.001^a^ |
| **Nodal stage** |  |  |
| N0 | 1(Ref.) |  |
| N1 | 1.20(1.09-1.33) | <0.001^a^ |
| N2 | 1.38(1.28-1.49) | <0.001^a^ |
| N3 | 2.29(2.11-2.48) | <0.001^a^ |
| **Stage** |  |  |
| S0-2 | 1(Ref.) |  |
| S3-4 | 1.64(1.52-1.76） | <0.001^a^ |
| **Chemotherapy** |  |  |
| No | 1(Ref.) | <0.001^a^ |
| Yes | 0.64(0.61-0.69) | <0.001^a^ |
| **Hormone therapy** |  |  |
| No | 1(Ref.) |  |
| Yes | 0.36(0.34-0.37) | <0.001^a^ |
| **Immunotherapy** |  |  |
| No | 1(Ref.) |  |
| Yes | 0.79(0.71-0.89) | <0.001^a^ |
| **Subtype** |  |  |
| Luminal | 1(Ref.) |  |
| Triple negative | 3.53(3.36-3.70) | <0.001^a^ |
| Her-2 | 1.56(1.42-1.70) | <0.001^a^ |
| **Surgery** |  |  |
| Simple mastectomy | 1(Ref.) |  |
| BCS/other | 0.97(0.91-1.04) | 0.452 |
| Radical mastectomy | 1.48(1.40-1.56) | <0.001^a^ |

Abbreviations: BCS, breast-conserving surgery.

^a^ The statistical tests were two-sided, the significance level was 0.05.
